# Screening and Functional Analysis of the Peroxiredoxin Specifically Expressed in *Bursaphelenchus xylophilus*—The Causative Agent of Pine Wilt Disease

**DOI:** 10.3390/ijms150610215

**Published:** 2014-06-10

**Authors:** Han-Yu Fu, Jia-Hong Ren, Lin Huang, Hao Li, Jian-Ren Ye

**Affiliations:** 1 College of Forest Resources and Environment, Nanjing Forestry University, Nanjing 210037, Jiangsu, China; E-Mails: fuhanyu2019@gmail.com (H.-Y.F.); renjiahong04@gmail.com (J.-H.R.); Lhuang@njfu.edu.cn (L.H.); lihao201404@gmail.com (H.L.); 2 Department of Biological Science and Technology, Changzhi College, Changzhi 046011, Shanxi, China

**Keywords:** *Bursaphelenchus xylophilus*, comparative proteomics, 2-DE, *in situ* hybridization, prokaryotic expression

## Abstract

The pine wood nematode, *Bursaphelenchus xylophilus*, is the causal agent of pine wilt disease. Accurately differentiating *B. xylophilus* from other nematodes species, especially its related species *B. mucronatus*, is important for pine wood nematode detection. Thus, we attempted to identify a specific protein in the pine wood nematode using proteomics technology. Here, we compared the proteomes of *B. xylophilus* and *B. mucronatus* using Two-dimensional gel electrophoresis (2-DE) and matrix-assisted laser desorption/ionization-time-of-flight/time-of-flight (MALDI-TOF/TOF-MS) technologies. In total, 15 highly expressed proteins were identified in *B. xylophilus* compared with *B. mucronatus*. Subsequently, the specificity of the proteins identified was confirmed by PCR using the genomic DNA of other nematode species. Finally, a gene encoding a specific protein (Bx-Prx) was obtained. This gene was cloned and expressed in *E. coli*. The *in situ* hybridisation pattern of *Bx-Prx* showed that it was expressed strongly in the tail of *B. xylophilus*. RNAi was used to assess the function of *Bx-Prx*, the results indicated that the gene was associated with the reproduction and pathogenicity of *B. xylophilus*. This discovery provides fundamental information for identifying *B. xylophilus* via a molecular approach. Moreover, the purified recombinant protein has potential as a candidate diagnostic antigen of pine wilt disease, which may lead to a new immunological detection method for the pine wood nematode.

## 1. Introduction

The pine wood nematode (PWN), *Bursaphelenchus xylophilus* (Steiner & Buhrer) Nickle, is the primary pathogen causing rapid wilting of *Pinus thunbergii* Parl. and *P. densiflora* Siebold & Zucc. in East Asia and *P. pinaster* Aiton in Portugal Europe, resulting in the deaths of millions of pine trees annually [1,2,3,4]. Correctly differentiating *B. xylophilus* from other species of nematodes in pines is the key to controlling and preventing the rapid spread of pine wilt disease.

However, identification of *B. xylophilus* is complicated by *Bursaphelenchus mucronatus* Mamiya & Enda [5]. *B. mucronatus*, a very similar species to *B. xylophilus* in morphology and biology, was discovered in the wood of dead pine trees and described as a new species [6]. It was reported that *B.* *mucronatus* has very low virulence or no pathogenicity to host pine trees compared with *B. xylophilus* [6,7,8]. Thus, differentiating these two species is crucial.

The traditional method of distinguishing these species is based on morphological differences. Mamiya and Enda (1979) [6] distinguished *B. xylophilus* from *B. mucronatus* according to its rounded tail shape with no distinct mucron. However, Wingfield *et al*. (1983) [9] found that female *B. xylophilus* from North America showed variations in tail shape from rounded to mucronated. Thus, identification of the two species using morphological characters alone may lead to misidentification [10,11,12]. Moreover, morphological detection is time- and labour-intensive.

Presently, serological techniques are the major means of detecting bacteria, viruses, and phytoplasmas and play a very important role in plant disease diagnoses and pathogen identification [13,14]. Lawler (1993) [15] used a serological technique to distinguish *B. xylophilus* from *B. mucronatus*. The results suggested that antiserum to *B. xylophilus* could distinguish the two species on Western blots, but that polyclonal antibodies did not distinguish the two species clearly using an ELISA system. This was due to the poor specificity of the polyclonal antibody. Thus, this method has not become popular.

To date, various molecular techniques have been developed for differentiating *B. xylophilus* from *B. mucronatus*, using polymerase chain reaction-restriction fragment length polymorphism (PCR-RFLP), random amplified polymorphism DNA (RAPD) techniques, species-specific primers based on internal transcribed spacers (ITS), and SCAR molecular marker, satDNA and *Hsp70* genes [16,17,18,19,20,21,22,23]. Recently, a methodology using loop-mediated isothermal amplification (LAMP) was developed for the direct detection of PWN [24]. However, these detection techniques mentioned are based on nucleic acids.

The major goal of comparative proteomics is to determine proteomic differences in the same species in different developmental stages or between allied species. As with many ecologically important species, proteomics research in *B. xylophilus* lags far behind that in other nematodes, such as *Caenorhabditis elegans* (Maupas) Dougherty and *Brugia malayi* Brug [25,26]. The surface coat proteins of the PWN expressed during host pine infection and *in vitro* culture have been compared using a proteomics approach [27]. The secretome of *B. xylophilus* was analysed by a proteomics method combined with the available genomic sequence. The study revealed the tangled roots of parasitism and the potential for molecular mimicry [28]. However, little research on the differential proteomics of PWN and the related species *B. mucronatus* has been reported. Moreover, few scholars have attempted to identify PWN-specific proteins to develop a detection method for *B. xylophilus*. Most genes in the genome achieve their function through protein expression, modification and interaction. Thus, the analysis of the proteins is important for investigation of the molecular mechanisms of the phenomena of life [29].

In the present study, to gain a better understanding of proteins differentially expressed between *B. xylophilus* and *B. mucronatus*, a proteomics approach was used to identify a specific protein of PWN. 2-DE coupled with MALDI-TOF/TOF-MS was used to separate and identify proteins from the nematodes. Protein spots that were specifically expressed by *B. xylophilus* with relatively high abundance were selected for a series of studies. The specificity of the differentially expressed proteins was confimed using PCR with the genomic DNA from other nematode species. Subsequently, *in situ* hybridisation was used to identify sites of expression. The gene encoding the specific protein identified was cloned and expressed. RNAi was used to evaluate the function of the *Bx-Prx* gene. The specificity of the *B. xylophilus* protein identified and the encoding gene will facilitate development of detection technologies for *B. xylophilus*.

## 2. Results and Discussion

### 2.1. Comparative Proteome Analysis and Protein Identification

**Figure 1:**
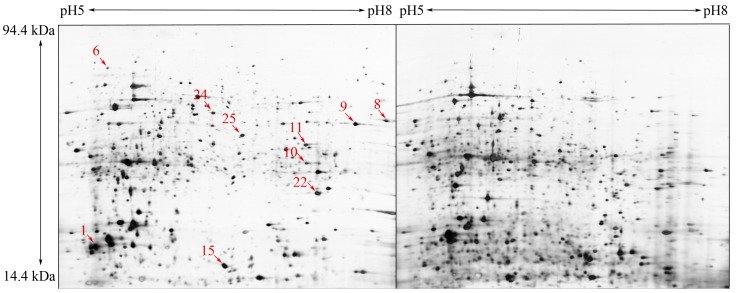
.Two-dimensional sodium dodecyl sulfate-polyacrylamide gel electrophoresis (SDS-PAGE) gels of *B. xylophilus* and *B. mucronatus* (**A**) *B. xylophilus*; and (**B**) *B. mucronatus* Proteins (120 µg) of *B. xylophilus* and *B. mucronatus* extracts were separated, in the first dimension by isoelectric focusing (pH 5–8) and in the second dimension by SDS-PAGE in 12.5% acrylamide gels. Proteins were visualised by silver staining. Proteins indicated by arrows were specifically expressed by *B. xylophilus* and identified by MALDI-TOF/TOF.

To identify PWN-specific proteins, we performed a comparative proteomics analysis. In this study, the focus was on *B. xylophilus* and *B. mucronatus*, which are closely related; however, the latter is considered to have very low virulence. Representative 2-DE gels of protein spots from the nematodes are shown in Figure 1. Approximately 2000 protein spots were detected on the 2-DE gel (Figure 1). The pattern revealed a broad distribution of spots in the pI range of 5 to 8 with an apparent molecular mass of 14.4–94.0 kDa. Examination of the 2-DE gels using the Image Master 2D Platinum 6.0 software (GE Healthcare, Uppsala, Sweden) revealed 35 protein spots with significant intensity differences, >1.75, between the two nematodes. Of them, 15 protein spots specifically expressed in *B. xylophilus* with relatively high abundance were excised from the gels and analyzed by MALDI-TOF/TOF. Database search results are listed in Table 1. The proteins identified, including actin, chaperonin Cpn60, GAPDH-1, aldolase, heat shock protein 70, aalectin-1, cytosolic fatty-acid binding, elongation factor 2, aldo/keto reductase, and peroxiredoxin, are involved in several processes, including cytoskeleton organization, protein folding, glycolysis, stress response, fruiting body development, transcription, ethanol oxidation and defense response.

The ability of proteomics to identify proteins is limited by the availability of sequence data [30]; thus the published genome data of PNW [31] are an important resource in the identification of proteins from this unusual parasite.

**Table 1:** Identification of specifically expressed proteins induced in *B. xylophilus.*

Spot No.	Accession No. ^a^	Protein Name	pI/kDa ^b^	Score ^c^	PN ^d^	Molecular Function
1	BUX.s00713.141	Actin	42/5.3	264	6	ATP binding, structural constituent of cytoskeleton
6	BUX.s00789.28	Chaperonin Cpn60	60/5.39	213	13	ATP binding
8	BUX_s01281.46	GAPDH-1	37/7.68	419	10	NAD+ activity
9	BUX.s01438.76	Aldolase	40/7.66	120	2	fructose-bisphosphate aldolase activity
10	BUX.s01653.149	Heat shock protein 70	71/5.55	528	14	ATP binding
11	BUX.s01109.344	Galectin	33/6.22	146	5	galactoside binding
15	BUX_s01226.18	Cytosolic Fatty-acid binding	88/6.14	71	9	transporter activity
24	BUX.s00397.100	Elongation factor 2	96/6.4	280	14	translation elongation factor activity
25	BUX.s01143.143	Aldo/keto reductase	37/6.22	40	3	oxidoreductase activity
22	BUX.s01109.415	Peroxiredoxin	22.1/6.09	156	6	thioredoxin peroxidase activity

^a^ Database accession numbers according to BUX.v1.2.genedb.protein.fa; ^b^ pI/kDa values were retrieved from the protein database; ^c^ Mascot score reported after searching against the geneDB database at: www.genedb.org/Homepage/Bxylophilus and ^d^ Number of peptides sequenced.

### 2.2. PCR Detection for Specificity

**Figure 2:**
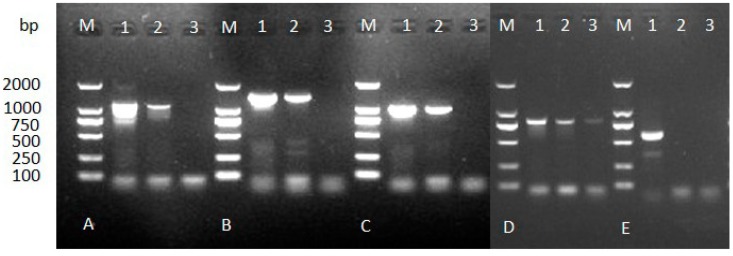
Amplified fragments of genomic DNAs of *B. xylophilus* and *B. mucronatus* using specific primers. (**A**): *aldolase*; (**B**): *actin-4*; (**C**): *elongation factor 2*; (**D**): *galectin-1* and (**E**): *thiol peroxiredoxin* (**M**): DL2000 marker; **1**, **2**, **3**, genomic DNA of *B. xylophilus*, *B. mucronatus* and ddH_2_O as templates, respectively).

**Figure 3:**
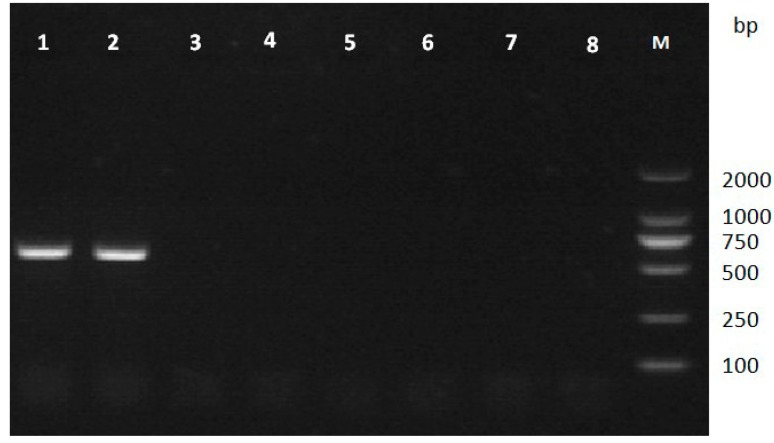
PCR amplification of genomic DNA of *B. xylophilus*, *B. mucronatus* and other nematode species using *Bx-Prx* gene-specific primers (Bx-PrxF1/R1). Only *B. xylophilus* had an amplification product at 749 bp. Lanes **1**–**2**, *B. xylophilus* strains; Lanes **3**–**5**, *B. mucronatus* strains; Lanes **6**–**8**, *B. hofmanni* (SF2), *Seinura wuae* (JY13), *Aphelenchoides macronucleatus* (GHHC2).

According to mass spectrometry results and the *B. xylophilus* genome data, five genes encoding actin (spot 1), aldolase (spot 9), galectin-1 (spot 11), peroxiredoxin (spot 22), and elongation factor 2 (spot 24) were successfully amplified from *B. xylophilus* genomic DNA by PCR (Figure 2). Subsequently, the specificity of the five genes was assessed by PCR using genomic DNA from other nematodes. The results suggested that only the gene encoding peroxiredoxin (Bx-Prx) was specific to *B. xylophilus* (Figure 3). Various strains of *B. xylophilus* yielded amplification products of 749 bp, while strains of *B. mucronatus* and other nematodes showed no amplification. Therefore, the *Bx-Prx* gene was unique at the nucleotide sequence level.

This might have been because the expression levels of the other four genes in *B. mucronatus* were below the detection limit of 2-DE. RT-PCR and quantitative real-time RT-PCR enabled identification of two genes expressed in a stage-specific manner (*AC-cathB-1*, *AC-cathB-2*) in *Angiostrongylus cantonensis* (Chen). *AC-cathB-1* and *AC-cathB-2* were expressed in L1 and L3, respectively, suggesting that *AC-cathB-1* and *AC-cathB-2* may play an important role in intermediate and final host invasion [32].

### 2.3. Expression, Purification and Identification of Bx-Prx

**Figure 4:**
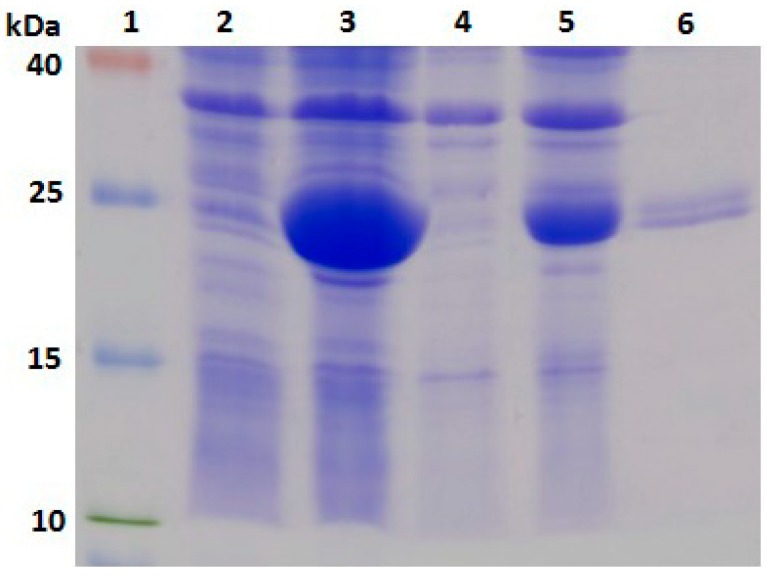
SDS-PAGE of the expressed product for pET 28a (+)-*Bx-Prx* in *E. coli* BL21(DE3). Lane **1**: molecular weight markers (40, 25, 15, 10 kDa); Lane **2**: soluble fraction of induced cells transformed by pET-28a; Lane **3**: soluble fraction of induced cells transformed by pET 28a (+); Lane **4**: inclusion body fraction of induced cells transformed by pET-28a and Lane **5**: inclusion bodies fraction of induced cells transformed by pET-28a (+)-*Bx-Prx*; Lane **6**: the purified pET 28a (+)-*Bx-Prx* recombinant protein.

**Figure 5:**
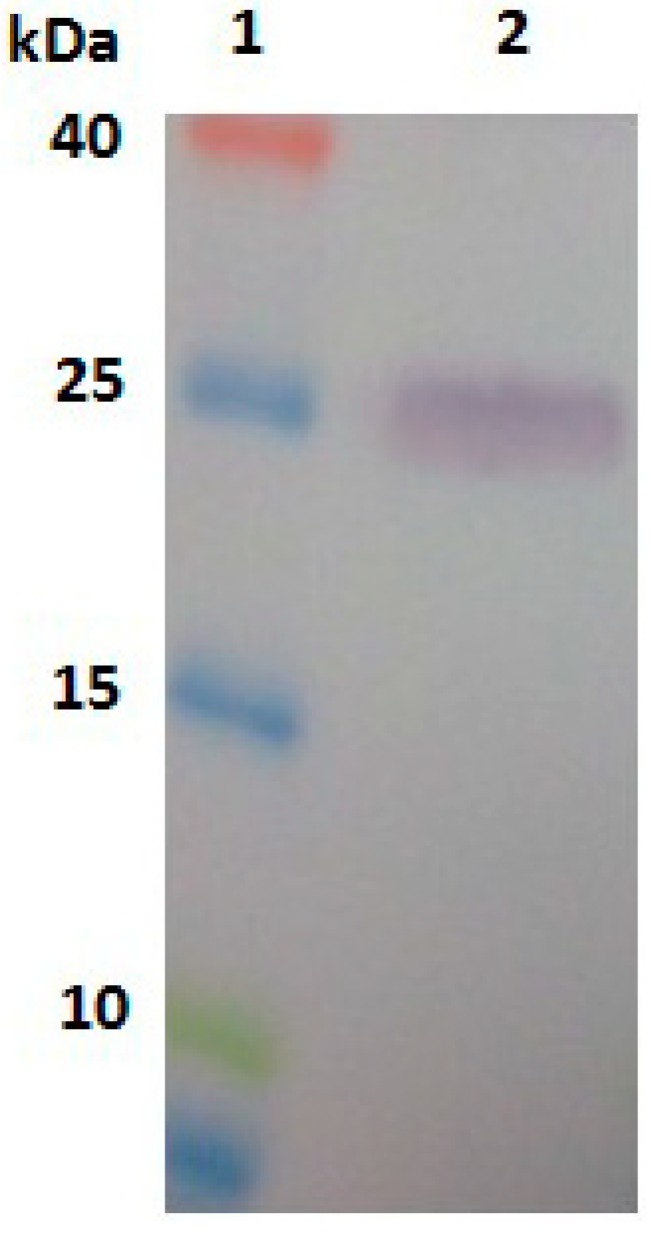
Western blots of protein purified from *E. coli* BL21(DE3) harbouring pET 28a (+)-Bx-Prx. Lane **1**: molecular weight markers (25, 15, 10, 4.6 kDa) and Lane **2**: Western blots of purified proteins.

To further analyse the Bx-Prx protein, the encoding gene was cloned and expressed in a prokaryotic expression system. Total protein samples from IPTG-induced *E. coli* cells were sonicated and separated by SDS-PAGE. The gel revealed a 26-kDa protein in the supernatant and pellet of bacteria harbouring *Bx-Prx*/pET-28a (+), consistent with the expected molecular weight of Bx-Prx. This result suggested that the recombinant protein exits *E. coli* in a soluble form and in inclusion bodies. No new band was evident in the control group (Figure 4). After nickel-nitrilotriacetic acid (NiNTA) purification, the purified recombinant protein was assessed by Western blotting using its 6× His sequence (Figure 5). A polyclonal antiserum against *Bx-Prx* recognized the target *Bx-Prx* as a single band, in only the extract from *B. xylophilus* not *B. mucronatus* [33]*.* Therefore, the purified Bx-Prx protein can be used as the candidate diagnostic antigen of pine wilt disease, facilitating development of immunological PWN detection methods. It was reported that two *Prx2* genes from the parasitic nematode, *Haemonchus contortus* (Rudolphi) Cobb was recombinantly expressed, whose result indicated that the *H. contortus* *Prx2* gene played an important role in hydrogen peroxide instability [34].

### 2.4. In Situ Hybridisation

**Figure 6:**
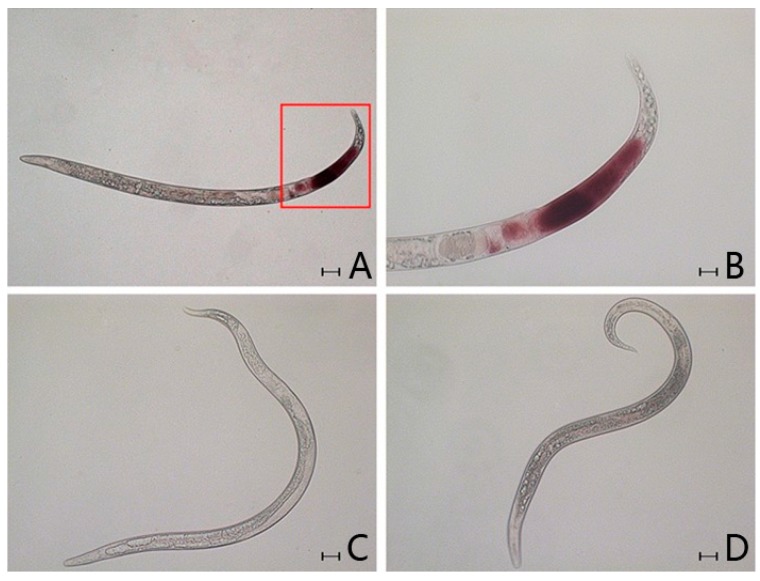
Localisation of Bx-Prx in the sexual gland. (**A**) *B. xylophilus*, hybridisation site was showed in the red table; (**B**) details of hybridisation signal of *B. xylophilus*; (**C**) *B. xylophilus* hybridised without a labelled probe and (**D**) *B. mucronatus*. Scale bars = 50 µm in (**A**,**C**,**D**) and 20 µm in (**B**).

*In situ* hybridisation (ISH) enables investigation of gene function in nematodes [35,36,37]. In this study, ISH was used to analyse expression patterns of the gene encoding the Bx-Prx protein. A digoxigenin-labeled probe generated from *Bx-Prx* specifically hybridised in the tail of *B. xylophilus* (Figure 6a,b). No hybridisation was observed in *B. mucronatus* (Figure 6d) or the control group (Figure 6c). Therefore, Bx-Prx was specific to *B. xylophilus* and apparently absent from *B. mucronatus*. The immunohistolocalisation study using a polyclonal antiserum against recombinant *Bx-Prx* suggested that the protein was localized under the cuticle of the nematode and in muscle cells and particles [33]. Thus, *Bx-Prx* is expressed under the cuticle of the nematode tail.

Superoxide dismutase, catalase, glutathione peroxidase and peroxiredoxin are major antioxidants in many organisms. The peroxiredoxins (Prxs) are ubiquitous thiol-specific peroxidases with catalytic functions in the detoxification of cytotoxic peroxides. The Prxs constitute a family of haem-free peroxidases with multiple functions. Their major functions can be divided into the following three categories: antioxidant activity by reducing alkyl peroxide and hydrogen peroxide to alcohol or water [38,39]; protection against phospholipid peroxidation [40] and protection of cells from oxidant-induced membrane damage and prevention of cell death [41]. The crucial role of peroxiredoxins in peroxide metabolism has been established in bacteria, plants and parasitic nematodes [34,42,43]. Most of these studies showed that the Prxs exhibited peroxidase activity or switched to a molecular chaperone upon heat shock or oxidative stress. Recently, relevant research on peroxiredoxins in the pine wood nematode demonstrated that the *Bx-Prx* protein plays an important role in protecting PWN against host immune responses [33]. Bx-Prx is one of 12 antioxidant proteins identified in the secretome of *B. xylophilus*. Futhermore, Bx-Prx was suggested to be basically a somatic protein and was also leaked from the PWN body. Given this background, we suggest that the secreted antioxidant Bx-Prx might play a role in protecting *B. xylophilus* from oxygen free radicals in the pine tree [28]. While *B. xylophilus* and *B. mucronatus* are closely related species, *B. mucronatus* has low virulence or no pathogenicity to host pine trees. Thus, the pathogenic differences between the species might be related to the differential expression of pathogenicity-associated genes. However, while our data suggests that *Bx-Prx* was expressed differentially between the two parasites; further studies are needed to determine the role of the *Bx-Prx* gene in parasite-host interactions.

### 2.5. Effect of RNAi on B. xylophilus Reproduction and Pathogenicity

RNA interference (RNAi) was first described by Fire *et al*. (1998) [44] in *C. elegans* for analysis of gene function *in vitro*. In this study, RNAi was used to assess the function of Bx-Prx in *B. xylophilus*.

**Figure 7:**
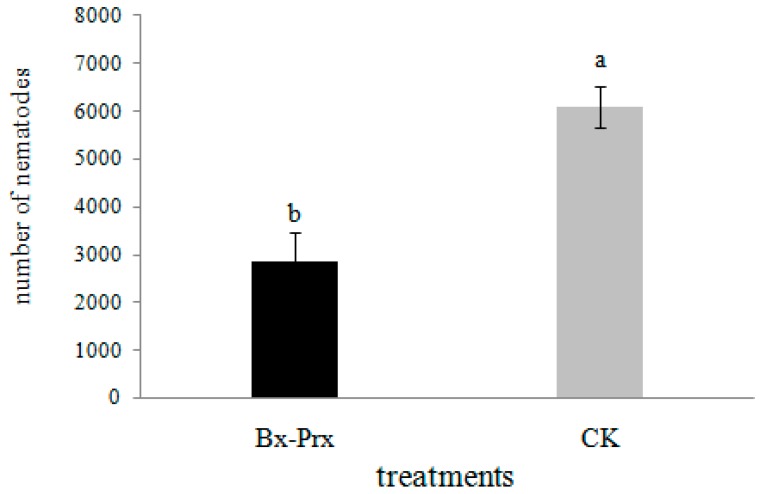
Propagation of *B. xylophilus* washed from the PDA plate of *B. cinerea* with and without dsRNA treatment. *Bx-Prx* and CK indicate the numbers of nematodes with and without dsRNA treatment, respectively. Bars show standard errors of the mean. The different letters on top of the bars (a,b) indicate statistically significant differences was found between the dsRNA-treated and controls (*p* < 0.05).

**Figure 8:**
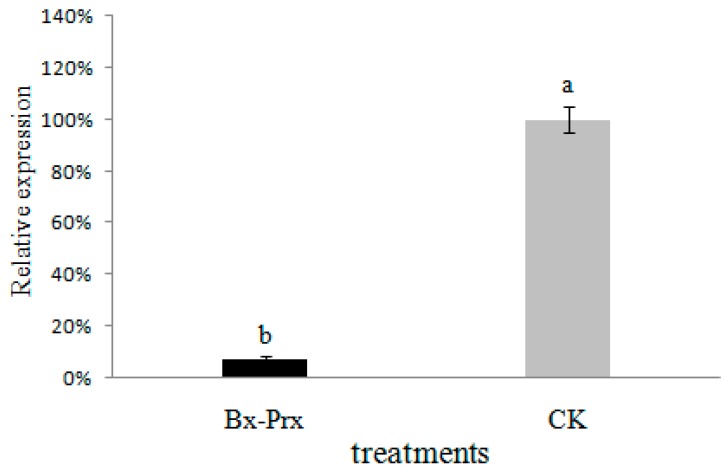
Bx-Prx mRNA levels in *B. xylophilus* after soaking in target dsRNA solution relative to the control (double-distilled water (DDW) with no dsRNA soaked). The expression level of the control was taken as 100%. Bars show standard errors of the mean. The different letters on top of the bars (a,b) indicate statistically significant differences was found between the dsRNA-treated and controls (*p* < 0.05).

The effect of RNAi on *B. xylophilus* propagation was tested on PDA plates inoculated with *B. cinerea*. The nematode soaked in double-strain RNA (dsRNA) solution showed significantly reduced propagation compared with the control. The number of nematodes in the dsRNA treatment was 2867 compared to 6100 after 7 days (Figure 7). These results indicated that feeding and reproduction of *B. xylophilus* were influenced by the RNAi treatment.

q-PCR was performed to determine the effect of RNAi on the *Bx-Prx* mRNA level. The *actin* gene of *B. xylophilus* was used as a reference gene. Soaking of nematodes in dsRNA solution resulted in a marked decrease in *Bx-Prx* gene expression compared to the control (Figure 8). Taking the mRNA expression level of the control as 100%, the mean expression level of dsRNA-treated samples was 0.071% (*p* < 0.001). These result suggest that knockdown of the *Bx-Prx* gene by RNAi is effective.

**Figure 9:**
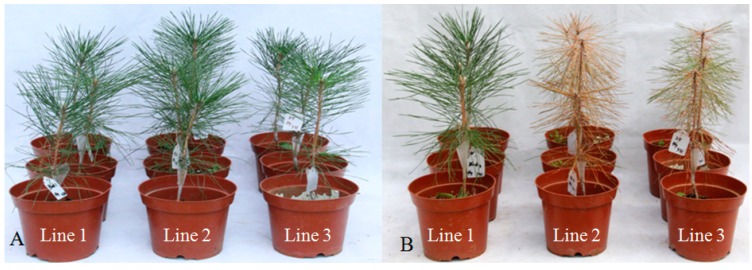
Symptoms in *P. thunbergii* seedlings 0 (**A**) and 30 days (**B**) after inoculation with nematodes soaked in dsRNA solution (Line **3**) and double-distilled water (DDW) (Line **2**). *P. thunbergii* seedlings inoculated with DDW alone was used as controls (Line **1**).

In 2-year-old *P. thunbergii* seedlings, inoculation with dsRNA-treated nematodes resulted in wilting and red-brown cones (Figure 9A). In contrast, only part of the plants inoculated with control nematodes wilted with some cones losing their green color (Figure 9B). These results indicated that *B. xylophilus* with down-regulated peroxiredoxin could result in a loss of pathogenicity.

Recently, RNAi has been applied to the investigation of arthropods [45], insects [46] and plant parasitic nematodes [47]. Moreover, RNAi has also been used to assess the pathogenic and molecular effects of the silenced *B. xylophilus* genes [48,49,50]. In this study, knockdown of the *Bx-Prx* gene markedly decreased the feeding, reproduction and pathogenicity of *B. xylophilus*. These results suggest that the *Bx-Prx* gene, knockdown of which results in the loss of pathogenicity, is important in PWN. It was reported that the RNAi efficiency by soaking may have worn off after a period of time [50,51,52,53], so further studies focus on the persistence of RNA interference are needed. However, the mechanisms by which it affects reproduction and pathogenicity will be addressed in future research.

## 3. Experimental Section

### 3.1. Experimental Organisms

*B. xylophilus* and *B. mucronatus* were isolated from *P. massoniana* by the Key Laboratory of Pest Prevention and Control of Jiangsu Province, Nanjing, China. The nematodes were cultured for 1 week at 25 °C on *Botrytis cinerea* Pers. grown on autoclaved barley grains and were separated from *B. cinerea* hyphae on a Baermann funnel for 6–8 h at 25 °C according to the method of Kikuchi *et al*. (2011). The nematodes were then washed five times in M9 buffer to remove any remaining *B. cinerea* mycelia, conidia, and hyphal fragments [31].

### 3.2. Protein Extraction

Proteins of a large number of nematodes were extracted according to the trichloroacetic acid (TCA)-acetone procedure described previously [54]. Briefly, nematodes were ground in a mortar with liquid nitrogen until a fine white powder was produced, and then suspended with 3 mL/g fresh weight ice-cold acetone containing 10% TCA and 0.07% dithiothreitol (DTT). The resulting protein-containing suspension was allowed to precipitate overnight at −20 °C and then centrifuged (20,000× *g*, 20 min). The pellet was rinsed three times with ice-cold acetone containing 0.07% DTT at −20 °C. Finally, the protein pellet was air-dried and dissolved in lysis buffer solution (8 M urea, 2% CHAPS, 0.05% immobilized pH gradient (IPG) buffer, 18 mM DTT). The protein concentration was measured using 2-D Quant Kit (GE Healthcare, Uppsala, Sweden) and the solution was aliquotted into 1.5 mL tubes for storage at −80 °C.

### 3.3. IEF and SDS PAGE

Each sample containing an estimated 120 µg of protein in 450 µL of IEF buffer was loaded onto a 24 cm immobilised pH 5–8 gradient strip (GE Healthcare Biosciences, Uppsala, Sweden). The strips were focused using a Ettan IPGphor Multiphor III (GE Healthcare, Uppsala, Sweden) at 20 °C, applying the following program: 30 V for 6 h, 60 V for 6 h, 200 V for 1 h, 500 V for 1 h, 1000 V for 1 h, 4000 V for 1 h, 8000 V gradient for 30 min, and 8000 V for 64,000 Vh. After focusing, proteins were reduced in equilibration buffer (6 M urea, 29.3% *w*/*v* glycerol, 2% SDS, and 1.5 M Tris-HCl, pH 8.8) containing 1% *w*/*v* DTT for 15 min, followed by alkylation in a separate incubation for an additional 15 min in equilibration buffer containing 2.5% *w*/*v* iodoacetamide (IAA) instead of DTT. The strips were then transferred to 12.5% SDS PAGE gels for 2-DE using Amersham’s Ettan DALT six gel system (GE Healthcare, Uppsala, Sweden) with SDS electrophoresis buffer (250 mM Tris, pH 8.3, 1.92 M glycine, and 1% *w*/*v* SDS) and 2 W/gel for 30 min and 17 W/gel for 5 h. At least three gels were run for each sample.

### 3.4. Image Analysis

Protein spots were detected by silver staining [55]. Stained gels were scanned and calibrated using the Labscan 5 software (GE Healthcare, Uppsala, Sweden). Three well-separated gels of each sample were used to create “replicate groups”. Detection and matching of the protein spots was facilitated with the use of the Image Master 2D Platinum 6.0 (GE Healthcare, Uppsala, Sweden) software and re-evaluated by visual inspection. Spots that were expressed specifically by *B. xylophilus* in relatively high abundance were analysed by matrix-assisted laser desorption/ionisation-tandem time-of-flight mass spectrometry (MALDI-TOF/TOF). The UniProt database (http://www.uniprot.org) was searched to determine the functions of the proteins identified. The peptide mass data were analysed for corresponding protein matching in the geneDB database. A positive identification had to meet the following criteria: a significant MASCOT score and at least four matched peptides in MS analysis or two matched peptides in MALDI-TOF/TOF analysis.

### 3.5. PCR Detection for Specificity of Differential Proteins

According to the results of mass spectrometry and PWN genome-wide data, five genes (primers used for gene cloning are listed in Table 2) were amplified from *B. xylophilus* genomic DNA by polymerase chain reaction (PCR). Briefly, specific PCR reactions were conducted in 20 µL volume, containing 2 µL primers mixture (10 µM), 1 µL DNA template, 10 µL ExTaq mixture and 7 µL sterile water. PCR reactions were performed with the following cycle conditions: (**a**) initial activation at 94 °C for 2 min; (**b**) 35 cycles of 94 °C for 2 min, 55 °C for 45 s, and 72 °C extension for 45 s; and (**c**) a final extension at 72 °C for 10 min. The amplified PCR products were confirmed by electrophoresis on 2% agarose gels. Gels were stained with ethidium bromide and viewed under a UV light. Then, the specificity of the five cloned genes was confirmed by PCR using genomic DNA from the other nematode species as the template.

**Table 2:** Identification of specificallly expressed proteins induced in *B. xylophilus.*

Gene	Spot No.	Sequence ID	Sense Primer (5-3)	Antisense Primer (5-3)
*Actin-4*	1	gb|ACZ13341.1|	ATGTGTGACGAAGAAGTTGCCGCTC	TTAGAAACATTTGCGGTGAACGATG
*Aldolase*	9	gb|ACZ13345.1|	ATGGCCGAAGTCGGTGCTTCTTATC	TTAGTATGCGTGGTCTACGAGAGGG
*Galectin-1*	11	gb|ACZ13331.1|	ATGACTGAGGAAAAGAAAACTTACA	TTAATGGATCTGGATGCCAGTGATC
*Elongation factor 2*	24	gb|ACZ13348.1|	CCGGAATTCATGAATCCTTCGGTTTCACCGCTG	CCCAAGCTTTTAGAGTTTATCGTAGAAGTTGTCG
*Bx-Prx*	22	gb|ABW81468.1	ATGTCCAAGGCTTTCATTGGCAAAC	TTAATGTTTGTTGAAATATTCGTGA

### 3.6. Expression, Purification and Identification of Bx-Prx

The entire open reading frame of *Bx-Prx* was cloned using *B. xylophilus* cDNA as the template and specific primers (5'-CGGGATCCATGTCCAAGGCTTTCATTGGCA-3' containing a *BamH*I site, and 5'-CGAGCTCTTAATGTTTGTTGAAATATTCGT-3' containing a *Sac*I site). The amplified products were digested with *BamH*I and *Sac*I (TaKaRa, Dalian, China) and ligated to the similarly digested pET-28a (+) vector using T4 DNA ligase at 37 °C. *E. coli* BL21 (DE3) cells were transformed by heat shock with the ligation product and plated on a selective medium. Subsequently, a single colony of the transformants was inoculated and cultured at 37 °C in LB medium containing kanamycin (50 µg/mL) with shaking (200 rpm) until the optical density (OD_600_) reached 0.6. Then, protein expression was induced by addition of isopropyl-β-d-thiogalactoside (IPTG) to a final concentration of 1 mM at 18 °C. Cultivation was continued for 20 h. Then, 200 mL bacterial culture was pelleted by centrifugation (8000× *g*, 10 min, 4 °C). Subsequently, the pellets were dissolved in Tris-HCl (20 mM, pH 8.0) and sonicated on ice. The supernatant and pellet were collected after centrifugation for a second time. The supernatant was purified based on its His-tag by affinity chromatography using HisTrap FF crude (GE Healthcare, Uppsala, Sweden). The soluble fraction, the insoluble fraction, the purified recombinant protein and the control (bacterium containing pET-28a (+)) were analysed on a 12.5% SDS-PAGE gel. Western blot analysis was conducted to confirm the recombinant Bx-Prx protein using an antibody raised against the poly-His tag (anti-His mouse IgG, Tiangen, Beijing, China).

### 3.7. In Situ Hybridisation

*In situ* hybridisation was performed on the *B. xylophilus* and *B. mucronatus* essentially as described previously [56,57,58]. Hybridisation probes were generated by amplification of the full length cDNA of Bx-Prx. PCR products were purified with the PCR Product Clean up Kit (Axygen Scientific, Inc., Union, CA, USA) and then random primer-labelled with digoxigenin using the DIG High Prime DNA Labeling and Detection Starter Kit I (Roche Applied Science, Mannheim, Germany). Then, the labelled probe was purified with the QIAquick Nucleotide Removal Kit (QIAGEN, Valencia, CA). *In situ* hybridisation was performed with mixed-stage *B. xylophilus* and *B. mucronatus* on poly-lysine-treated microslides. Nematodes were pre-treated with proteinase K (10 µg/mL) for 30 min at 37 °C before the post-hybridization washing step. Hybridisation and detection were performed with the DIG-High Prime DNA Labelling and Detection Starter Kit I (RocheDiagnostics, Mannheim, Germany), and examined using a Zeiss Axio Image M2 microscope (Zeiss MicroImaging GmbH, Oberkochen, Germany).

### 3.8. Bx-Prx Knockdown by RNA Interference (RNAi)

The nematodes were ground in a mortar with liquid nitrogen until a fine white powder was produced. Total RNA was extracted from the resulting powder with Trizol reagent (Invitrogen, CA, USA). The RNA was then reverse-transcribed using a cDNA synthesis Kit (TransGen Biotech, Beijing, China), according to the manufacturer’s protocol. Double-stranded RNA (dsRNA) was synthesised using the MEGscript RNAi Kit (Ambion Inc., Austin, TX, USA) with the primer set (Bx-Prx) designed in Section 2.5. The RNAi soaking method was performed basically according to Urwin *et al*. (2002) [59]. Freshly cultured nematodes of *B. xylophilus* (a mix of adults and juveniles, approximately 3000 individuals) were soaked in 30 µL dsRNA solution and incubated at 180 rpm for 24 h at 20 °C. *B. xylophilus* soaked in DDW without dsRNA were used as the negative control. After soaking, samples from each treatment were washed thoroughly several times in DDW and then used for further experiments. First, 15 pairs of female and male nematodes were picked and transferred onto a potato dextrose agar (PDA) plate with *B. cinerea* and cultured at 25 °C for 7 days. Then, the worms were washed from the plate and the nematodes were counted. Second, approximately 200 nematodes were used to verify the efficacy of Bx-Prx knockdown by qPCR carried out using a Kit (TransStart Green qPCR SuperMix; TransGen Biotech, Beijing, China) and a thermal cycler (ABI Prism 7500, Applied Biosystems, Foster City, CA, USA), with the following primers for Bx-Prx: 5'-GCTTTCCGTGGTTTGTTC-3' and 5'-ACTTCTCCGTGCTTGTCG-3' (from nucleotides 367–506). The cycling profile used was: 94 °C for 30 s, followed by 40 cycles of 94 °C for 5 s; and 60 °C for 34 s. *Actin* was used as a reference gene. Initial data analysis was performed using the ABI Prism 7500, which calculated *C*_t_ values and extrapolated relative levels of PCR products from standard curves. The 2^–ΔΔ*C*t^ method was used to quantify the relative changes in gene expression. qPCR was conducted for three biological replicates, which included three technical replicates per reaction. Finally, the remaining nematodes were subjected to pathogenicity determination according to a previous method [60] with inoculation onto 2-year-old *Pinus thunbergii* seedlings. Thus, 1000 nematodes were injected into each seedling. *B. xylophilus* soaked in DDW without dsRNA and DDW alone, were used as controls. Periodically, we observed the seedlings and took photographs to record their state. Three biological replicates were conducted.

## 4. Conclusions

In conclusion, using proteomic technologies, we identified a specific *B. xylophilus* protein encoded by a gene, which is apparently present only in *B. xylophilus*. The identification of this gene, cloned and expressed in *E. coli*, provides fundamental information for identifying *B. xylophilus* via molecular approaches. *In situ* hybridisation pattern of *Bx-Prx* showed that it was expressed in the tail of *B. xylophilus*. RNAi was used to evaluate the function of *Bx-Prx*, the results indicated that the gene was associated with the reproduction and pathogenicity of *B. xylophilus*. This discovery provides fundamental information for identifying *B. xylophilus* via molecular approaches. Furthermore, the purified recombinant protein has potential for use as a candidate diagnostic antigen of pine wilt disease, which may lead to development of a new immunological detection method for PWN.
